# Clinical Observations on the Efficacy of Hydro-Chloruret of Lime, as a Remedy in Certain Stages of Fever and Dysentery

**Published:** 1828-01-01

**Authors:** 


*Clinical Observations on the Efficacy of Hydro-Chloruret of Lime, as a Remedy in certain Stages of Fever and Dysentery.* By ROBERT REID, M.D. &c. &c. Octavo. Dublin, April, 1827.

IN the late—and, indeed, present epidemic fever of Dublin, (April, 1827,) Dr. Reid had occasion to notice some circumstances, not observed by himself or others in former epidemics. In individuals affected with the prevailing fever, the disease usually increased till the sixth day, when a severe rigor took place, followed or not by profuse perspiration. When this occurred, the fever was generally at an end, and the patient became convalescent—the speedy convalescence being generally proportioned to the severity of the rigor. The great majority of patients, however, had not this good luck. The disease yielded with difficulty to the usual remedies, and the patients were brought to a doubtful convalescence, thinking themselves well. There would be no tangible or appreciable disease—and yet the physician would frequently be surprised at hearing of his patient's sudden death the day after he had left him apparently convalescent.

We shall notice one or two cases in illustration. Anne Watson, aged 19, after being five weeks ill with fever, was received into the hospital on the 9th Jan. 1827. The disease seemed mild—her bowels were regulated by aperients—and she was then ordered the saline diaphoretics. She appeared gradually getting better till the 13th, when, after a quiet sleep, she awoke, gave a sigh, and died.

*Dissection.* The stomach was much distended with flatus, and some dark spots were observed on the mucous membrane. The duodenum and other intestines were natural. The liver not enlarged, but the colour of a deeper red than usual—"the biliary function apparently suppressed." There was much red fluid in the pericardium—left ventricle of the heart flabby, its parietes thin, and the cavity much larger than natural. "On opening this cavity, a gush of air rushed out, and it did not contain any blood or coagulum." The left auricle, on pressure between the fingers, gave a distinct sensation of crepitus, from air contained in its cavity. There was no sign of putrefaction in any part of the body. The right auricle was distended with venous blood; right ventricle natural. The contents of the pulmonary veins did not appear to have undergone the usual change in the lungs, which, in other respects, seemed healthy. The internal surface of the aorta was of a dull pink colour, and the texture of the vessel appeared softened—which state seemed to extend through the whole arterial system. In the head, the dura mater was found extremely vascular, and an extravasation of blood and coagulated lymph was observed covering the entire right hemisphere of the brain, causing a considerable depression in the middle region. The coagulated lymph pointed, as usual, to the source of the hæmorrhage, which was ascertained to have flowed from a small vessel at the side of the longitudinal sinus. There was no extravasation on the left hemisphere. The medullary substance of the brain seemed in a state of congestion.

*Case* 2. Catherine Hannon, a nurse of the hospital, took the fever, and was confined to bed, on the 9th March, 1827.

On the 12th, she appeared nearly convalescent. On the 13th, in the morning, she had a slight rigor, and in eight hours afterwards she was dead.

*Dissection.* The surface of the body exhibited a dirty purple yellow hue. The viscera of the thorax, abdomen, and pelvis, healthy. When the scull-cap was removed, the dura mater seemed of a brownish yellow colour, and a yellow matter, as if bile had been suffused, was found between the arachnoid and pia mater. The substance of the brain had a yellow tinge. Nothing remarkable in the ventricles.

*Case* 3. William Ludlow was admitted into the hospital, labouring under a relapse of fever. He seemed progressively mending until the 28th of March, when he complained of slight pain in the abdomen, and general feeling of returning disease. These symptoms rapidly increased, and next morning he died.

*Dissection.* The serous surface of the peritoneum was healthy—the mucous membrane of the ileum exhibited patches of congestion, with some tendency to ulceration. The viscera of the thorax and abdomen were healthy. The blood appeared decomposed and watery. The medullary substance of the brain was congested, and much watery blood was discharged from the cut parts.

*Case* 4. John Anderson was admitted on the 2d of April, being six days ill with fever: he complained of tenderness of abdomen on pressure—skin and eyes yellow. On the 5th he appeared much relieved, and had a quiet preceding night. At mid-day the pain of abdomen returned, and leeches were ordered; but, before any means could be used, he turned quite purple, and quickly died. There was no dissection.

*Case* 5. Master ——— had been ill several days with fever, but became convalescent. In a few days more he complained of violent pain in the abdomen, and leeches were ordered, but, from mistaken lenity, they were not applied. In 48 hours he died.

*Dissection.* Great tendency to putrefaction in a few hours after death—abdomen tumid, and integuments green—back and lower extremities black. On opening the abdomen all the viscera were found covered with a yellow curd-like matter, and a considerable quantity of pus and sanies diffused through the cavity. The liver was studded with a crystaline miliary eruption, similar to what had been observed on the surface during his illness. The head was not examined, as it had shewn no symptom of disorder during life.

"To avoid the farther detail of particular cases, it may be sufficient to state, that in all the subjects I have examined, who have died of the fever at present under consideration, there have been evidences of the maturation of the disease, modified, of course, by the nature of the parts upon which its influence was directed, and by the accidental state of the individual's constitution. Thus it was sometimes thrown on the vascular system, as in the first case, and by injuring the texture of the vessels, disposed them to rupture, or gave origin to hemorrhage from the nose and rectum. When the result of the disease has fallen on the substance of the brain, this organ after death exhibited a most remarkable state of congestion. On cutting the medullary mass, a quantity of thin blood, apparently in a dissolved state, was poured from innumerable points. These cases were by far the most rapidly mortal; some have occurred to my observation when this state of the brain was about to commence, and before many minutes elapsed, the progressive changes towards death took place while I was standing by the patient. In all the cases of this kind which I have examined, there were observed patches of several inchesi n extent of a dark colour upon the ileum. Upon opening this intestine these patches were found to be occasioned by a state of vascular congestion on the mucous surface, sometimes even apparently abrasions were observed; indeed, in one case there were several perforations, so that the contents of the intestine escaped into the cavity of the peritoneum. In these cases the fatal train of symptoms generally commenced with abdominal pain, but the state of the brain soon rendered the patient insensible to the progress of disease in the abdomen, although he may be still capable of giving a rational reply to the questions of his medical attendant.

"When the patients have survived a day or two after the result of the disease had been deposited on some internal part, bands of adhesions were observed; but in the rapidly fatal cases, the coagulable lymph and curd-like substance could be wiped off the organ, and then the surface would appear quite sound." 11.

From these and other circumstances, our author is induced to think that there is some peculiarity in this fever which disposes to the formation of a morbid matter that acts like a poison, and quickly destroys the powers of life. In this view, he is supported by Dr. Marsh, whose paper will be found reviewed in the present number of our Journal. In former epidemics, our author had observed many symptoms which seemed to indicate a tendency to generate a morbid poison; but he did not meet with the regular train of symptoms in any individual case, such as occurred in the present epidemic.

"The generation of this morbid poison appears to take place at some uncertain period of the disease, and seems as if thrown on particular organs or parts, exclusively leaving all other parts of the body apparently in health. Probably the peculiar state of the patient's constitution at the time, may determine the situation where the morbid change may be developed." 16.

Impressed with the idea of such a tendency in the prevailing fever, our author sought to counteract it, and, for this purpose, tried the hydro-chloruret of lime—first with patients who had no prospect of surviving their disease under the ordinary mode of treatment. The following is an instance.

*Case* 5. John Coyne was admitted into the hospital in the last stage of dysentery, which came on after a tedious fever. The discharges consisted of a bloody sanies, intermixed with a dark-coloured matter highly offensive. The stools were passed involuntarily, and the patient was reduced to an idiotic state. Dr. R. considered that erosion of the intestines had taken place, and that death was inevitable. He directed ten grains of the hydro-chloruret of lime to be added to the common enema of the Pharmacopœia, and to be thrown up, night and morning. The stench was corrected, the evacuations became more natural, and pure blood was sometimes discharged. His tongue became clean and moist—but exhaustion increased, yet he lived a fortnight after this. No dissection was made, and we believe that Dr. R. was right in supposing that the intestines were ulcerated, and that death was inevitable.

6. Encouraged by the effects of the hydro-chloruret in correcting the fetid discharges in the above patient, our author next directed its administration to a female lying ill with dysentery after fever, and whose evacuations were so intolerable, that the other patients declared they could not stay in the ward. The first day after the injections, the patient experienced considerable relief of all the symptoms. In a few more days, the fetor was corrected. The progress of the cure was gradual and steady, and she entirely recovered.

*Case* 7. In this case the medicine was exhibited by the mouth. The patient was a very old man, of broken constitution, who was admitted for dysentery succeeding fever. He had frequent bloody evacuations, attended with great pain in the abdomen—tongue loaded—thirst urgent—great debility—some appetite. Dr. R. directed a mixture composed of ten grains of hydro-chloruret of lime, two drachms of tincture of colombo, and four ounces of water, to be taken in half-ounce doses every hour. Next day the tongue became moist, though still loaded. The medicine to be continued. On the third day the evacuations were changing to a more natural appearance, and he had longer intervals from purging and pain. The medicine was continued for a few days longer, when he got quite well.

*Case* 8. This was a very formidable one. The patient had been in Sir Patrick Dunn's Hospital for dropsy, where he was tapped. A week after leaving the hospital, he was seized with dysentery, in which state he had been three weeks before he came into the Fever Hospital. He complained of severe pain in his bowels—had incessant alvine evacuations of thin fetid matter intermixed with bloody sanies. The abdomen was swelled, and his legs were œdematous. Two days were spent in the administration of some medicine, and then the hydrochloruret was given, nearly in the manner above related. He soon began to mend, and, in a fortnight, the dysentery had greatly abated, and he was able to sit up and put on his clothes.

The above cases were selected from a great number of the same kind. Dr. R. thinks they were cases that would, most probably, have terminated fatally under other treatment. Several cases are next detailed, in which the medicine was administered in certain stages of fever, and apparently with considerable advantage. These we need not state. The foregoing observations will probably lead many of our readers, in this and in other countries, to try the means recommended by Dr. Reid. We shall conclude this paper with the following extract, which also concludes the pamphlet.

" It may be necessary to state that the substance known in the shops under the name of hydro-chloruret of lime, is extremely variable as to the quantity of the soluble salt it contains. When first I employed this substance as a medicine, I prescribed the supposed necessary quantity of the dry substance. I soon observed, however, that the operation of the medicine appeared extremely irregular; I was, therefore, induced to examine the substance by some test which would give an indication of the quantity which was capable of being dissolved in water. As it was known that this substance has the property of discharging the colour of indigo when dissolved in sulphuric acid, I directed three grains of indigo to be dissolved in three drachms of sulphuric acid, to which were added three ounces of water. This I reserved as a standard of comparison. Three ounces of dry chloruret of lime having been put into a pint of water, the mixture was occasionally stirred for twenty-four hours, and then filtered through paper. The strength of this liquid was ascertained by the number of drops required to discharge the colour of twenty-five drops of the indigo solution. I observed that the strength of the liquid varied from nine to twelve. It was, therefore, directed that each quantity of the solution, on being filtered, should be marked so as to indicate the strength, or the number of drops capable of discharging the colour from twenty-five of the indigo solution.

" I have selected the foregoing cases from a great number, as being those in which the evidences of the efficacy of the hydrochloruret of lime were most unequivocal. From attentive observation relative to the mode of action of this remedy, I am induced to consider it astringent in its primary local action, without apparently exciting inflammation. In certain stages of dysentery, therefore, it will be found of the highest importance. The cases of Rose Macnamara and Heron evince its powerful efficacy in that stage of the disease which has been heretofore so tedious and intractable. As to its secondary influence, it seems to produce very general effects upon the functions of the ganglionic system, principally evident in correcting the formation of morbid matter. In some cases it appears to act as a diuretic. The urine, however, when increased in quantity under its influence, appears to be of that kind which was formerly supposed to indicate a termination of disease, thus agreeing in its effect with the observation of Hippocrates, '*cocta non cruda sunt evacuanda.*' More frequently perspiration seems to be its most evident effect. In such case also the same observation is fully applicable, as the perspiration is seldom profuse, but always of that healthy feel, which has been usually considered by the physician as the most satisfactory solution of a severe disease.

" From the observations I have made of the efficacy of this medicine, in cases which exhibited all the symptoms of that severe disease which medical writers have denominated yellow fever, I can with some confidence recommend it as a valuable remedy. Indeed, the cases to which I at present allude, having had all the symptoms of that formidable disease, which the difference of climate in these countries could admit, I am induced to expect, that when properly employed, the hydrochloruret of lime will be found as valuable a remedy in the treatment of yellow fever, as mercury has proved in syphilitic disorders.

" When a remedy has been found very efficacious in some states of disease, the practitioner, by not carefully observing its operation in the animal economy, is induced to appropriate the medicine rather to the name of the disease as known to nosologists, than to the morbid actions which the remedy is peculiarly adapted to correct.

" The practitioner, therefore, should be careful not to expect too much from any single remedy, for the records of medicine show how many substances have been at times lost to the physician, and at other periods proving of the highest value in the science of medicine." 35.

